# Genetic Variation in Insect Vectors: Death of Typology?

**DOI:** 10.3390/insects9040139

**Published:** 2018-10-11

**Authors:** Jeffrey R. Powell

**Affiliations:** Yale University, 21 Sachem Street, New Haven, CT 06520-8105, USA; jeffrey.powell@yale.edu

**Keywords:** typology, population thinking, vector biology, vector control, *Aedes aegypti*, *Anopheles gambiae*

## Abstract

The issue of typological versus population thinking in biology is briefly introduced and defined. It is then emphasized how population thinking is most relevant and useful in vector biology. Three points are made: (1) Vectors, as they exist in nature, are genetically very heterogeneous. (2) Four examples of how this is relevant in vector biology research are presented: Understanding variation in vector competence, GWAS, identifying the origin of new introductions of invasive species, and resistance to inbreeding. (3) The existence of high levels of vector genetic heterogeneity can lead to failure of some approaches to vector control, e.g., use of insecticides and release of sterile males (SIT). On the other hand, vector genetic heterogeneity can be harnessed in a vector control program based on selection for refractoriness.

## 1. Typological and Populational Thinking

There are two fundamental ways to view the biological world: As sets of nearly identical essential types (typology) or as populations of heterogeneous individuals (populational thinking). Typologists see the world as consisting of homogeneous populations in discrete groups with an idealized essence embodied by “normal” (wild type) individuals. The population view considers the living world to be composed of heterogeneous populations of unique individuals that can be assigned often only loosely to discrete groups or types. These nearly diametrically opposed views determine how research is designed and carried out and how data are interpreted and valued. While these views can be traced back to ancient Greece (e.g., Plato’s idealized types), we need only start around 1900 to appreciate how these views impact biology in general and vector biology in particular.

In 1900, Mendel’s principles of inheritance were rediscovered followed by the development of the field of genetics primarily by Thomas Hunt Morgan. An essential principal of genetics in this early stage was the recognition of a wild type and mutants. The wild type was the normal genotype that defined the species and predominated in all populations. Mutants were rare anomalies useful in genetic studies, but otherwise having little to do with the essence of the species. Morgan understood his genetic studies were important to understanding evolutionary processes and, in fact, wrote three books on the subject [1,2,3]. Morgan’s view of evolution was that it was driven by mutation. Most of the time, populations are composed of individuals all homozygous for wild type alleles. Rarely, a new favorable mutation occurs that quickly goes to fixation and a new set of homogeneous wild type is established. Morgan gave little role to natural selection except to weed out the unfit (discussed further in [4]). This mutation driven view of evolution persists [5].

Morgan’s books had little impact on the field of evolutionary biology primarily because they did not resonate with biologists who worked with natural populations (field biologists, taxonomists, ecologists, etc.). Such workers observed ample variation among individuals and populations in nature for a variety of evolutionarily important traits like morphology and behavior. Variation reigned rather than idealized types. For some time these workers doubted that the genetics as developed by Morgan and his school had much to do with what happens in real populations in nature. A rapprochement or sorts arose with the demonstration by R. A. Fisher, J. B. S. Haldane, and S. Wright in the 1920s and 1930s that, theoretically, Mendelian rules of inheritance were compatible with naturally occurring variation and Darwinian evolution. Dobzhansky [6] demonstrated how these mathematical theories are consistent with observations in natural populations. In contrast to Morgan’s mutation driven view of the evolutionary process, in this view, most evolutionary change is based on pre-existing genetic variation. Enough genetic variation is floating in natural populations for almost all traits to make adaptive evolutionary changes in the absence of any new mutations. Success by plant and animal breeders in selecting a plethora of diverse traits in many plants and animals confirmed this view. Population thinking began to replace typological thinking at least among those interested in natural history, ecology, and evolution, that is, workers interested in populations and species *as they exist in nature*, in contrast to laboratory cultures sitting on a shelf or in an incubator.

While population thinking was sweeping the world of ecology and evolution in the mid-20th Century, molecular biology was exploding as a discipline and most practitioners implicitly or explicitly embraced typology. T. H. Morgan was the intellectual father of many early molecular biologists. Biological problems could be reduced to a basic essence for which a physical/chemical explanation must hold across all of life. A popular quip at that time was “…anything found to be true for E. coli must also be true of Elephants” [7]. As R. C. Lewontin [8] pointed out, the two approaches to biology had fundamentally different goals and priorities. The molecular biologist sought *constancy*, general principles that held across the living world. Natural historians and population biologists consider *variation* to be the defining essence of biology, the basis of evolutionary change, and thus of all life. 

## 2. Genetic Heterogeneity in Vectors

The problems inherent in vector-borne diseases can be fruitfully considered a problem in ecology and evolution, or more generally, population biology. Vector-borne diseases are most often a three species interaction problem: The vector (usually an arthropod), the pathogen (usually a microbe), and the vertebrate host (humans in cases of most interest to us). The population dynamics and interactions of three species need to be considered simultaneously. And, equally importantly, each level is heterogeneous, i.e., consisting of a heterogeneous grouping of individuals. Here I will focus on the vector side with the explicit caveat that this is only one-third of the overall problem; heterogeneity of pathogens and humans (or other vertebrate hosts) are equally crucial in reaching a full understanding of these diseases.

**Figure 1:**
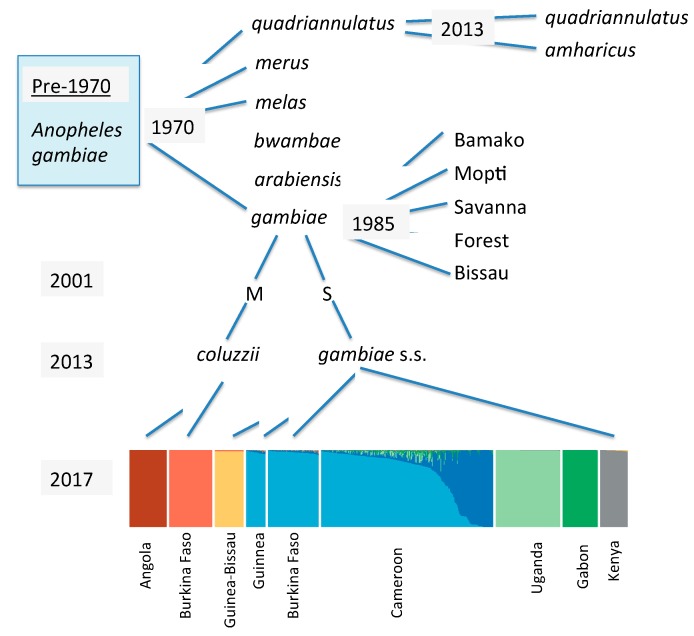
Schematic history of the taxonomy and genetic diversity of *Anopheles gambiae*. The light blue box in upper left indicates it was considered a single species up to the 1960s. Italicized names are formally described species; non-italicized designations are informal “forms”. The bottom colored block is a STRUCTURE plot [10] based on whole genome sequencing. Summarized from work reported in [11,12,13,14,15].

As just one prominent example of the importance of understanding the genetic heterogeneity of vectors we can consider the well-known and extremely important case of *Anopheles gambiae*, the primary vector of malaria in Africa where most deaths due to this disease occur. Mario Coluzzi and his collaborators studied chromosomal (inversion) polymorphism in this complex of species and uncovered a plethora of heterogeneity not distinguishable on the morphological level. Figure 1 is a quick historical overview of knowledge of this vector. What was considered a single species is now eight formally described species. In addition, various chromosomal forms were described [9], “form” being genetically distinct entities, but ambiguous with regard to taxonomic status. Furthermore, when two of the named species are examined in the detail provided by genome sequencing, individual populations are often found to be genetically distinguishable and unique [10]. And this is for *inter*-population variation. By far the most genetic variation exists *within* populations with nucleotide heterozygosity in these populations being in excess of 1% [10], i.e., an individual is heterozygous at >1% of all nucleotide sites which means that *no two individuals are genetically identical*.

**Figure 2:**
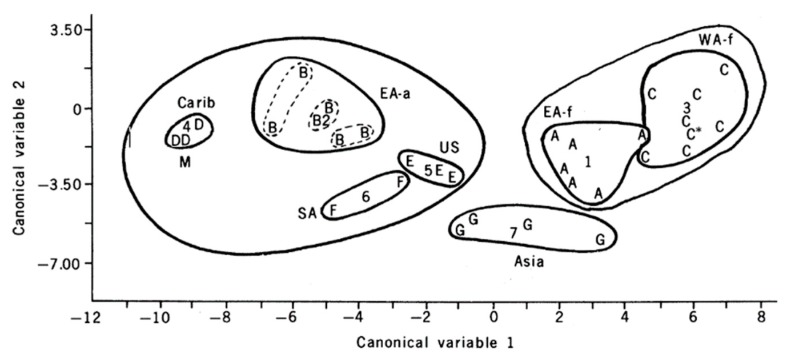
Step-wise linear discriminant analysis based on allozyme variation in populations of *Aedes aegypti*. Letters indicate population samples, numbers the center of lettered groups. A: East Africa formosus subspecies, B: East Africa aegypti subspecies, C: West Africa formosus, D: Caribbean, E: United States, F: South America, G: Asia. From Reference [19].

**Figure 3:**
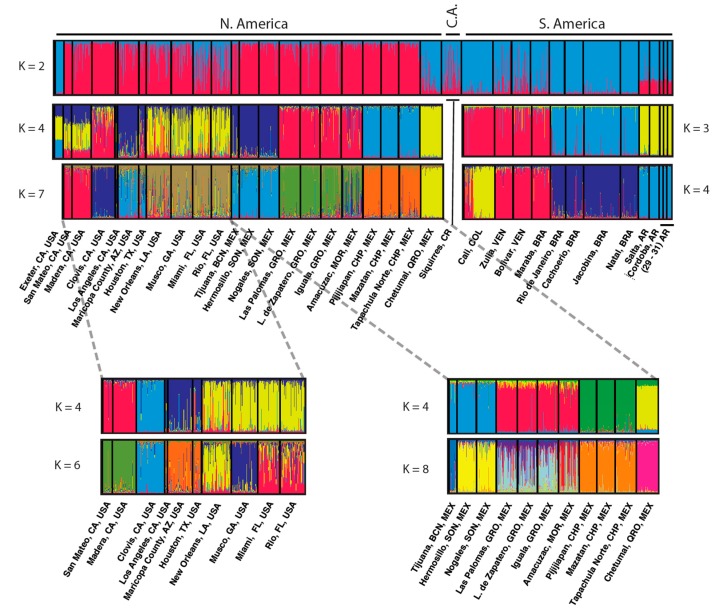
Genetic structure of population samples of *Aedes aegypti* from the Americas based on microsatellites. Bayesian STRUCTURE plots [10] are shown for different groups of populations. K is the number of subpopulations assumed. From [20].

A similar story holds for another well-studied vector, *Aedes aegypti*. This species is composed of two named sub-species that differ in morphology and behavior with a darker zoophilic form (*Ae. aegypti formosus*) occupying the ancestral sub-Saharan Africa region and a light anthropophilic form (*Ae. aegypti aegypti*) that has colonized the tropical and subtropical world outside Africa [16,17]. Even with the relatively crude method of electrophoresis of proteins, this species was found to be genetically differentiated into discrete populations throughout its distribution, Figure 2 [18,19]. More recent DNA-based technologies have only led to confirmation and further resolution of the discrete genetic patterns exhibited by this vector (Figure 3). While not every population is genetically distinct using these genetic tools, clearly the genetic diversity across populations is remarkable.

It is not only genetic variation detectable by molecular technologies that is ubiquitous in vectors. Chromosomal inversion polymorphisms are well known especially in anopheline mosquitoes with favorable polytene chromosomes. As noted earlier, M. Coluzzi’s work on *An. gambiae* is a monumental demonstration of the importance of inversion polymorphism in vectors (see references in [21]). In an early work, Craig et al. [22] showed that strains of *Ae. aegypti* from different localities varied in sex-ratio from about 22% to 52% females. Inbreeding revealed about 40 different morphologically identifiable mutations and that each of 30 strains examined carried on average one recessive morphologically identifiable mutation. McClelland [23] documents extensive morphological variation in the patterns of scales on *Ae. aegypti* both within and between the named subspecies. Jupp et al. [24] documented considerable variation among S. African samples of *Ae. aegypti* for the scaling patterns used to distinguish the subspecies, so much so that offspring of single wild-caught females displayed variation ranging from “pure” *Ae. aegypti formosus* to “pure” *Ae. aegypti aegypti*. This seriously questions the validity of the typological subspecies designations.

## 3. Importance of Variation Vector Biology

I will present four contexts in which heterogeneity of vector populations are important in vector biology. The first is how the heterogeneity of vectors is reflected in the relative importance of different subgroups and genotypes in transmitting diseases. The second, linkage disequilibrium, might seem a basic property of the genetic structure of populations, but it has an important use in understanding the genetic basis of many traits of particular interest to vector biology. The third context is determining the origin of new introductions of invasive species that continue to spread today. Finally, I discuss the difficulty in inbreeding vectors in attempts to reduce genetic variation, implying that much of the standing genetic variation in vectors contributes to their fitness and adaptive flexibility.

*Vector competence*: Vector competence, the relative efficiency with which a vector can acquire a pathogen and transmit it, is a fundamental factor in understanding any vector borne disease. This trait displays considerable variation among species, populations, and individuals. A few illustrative examples are presented here; Tabachnick [25] should be a consulted for a more detailed authoritative review of this issue.

On the species level, we can again use the example of *An. gambiae* complex. Some members of the complex (*An. gambiae* s.s., *An. arabiensis*, and *An. coluzzii*) are extremely efficient carriers of human malaria parasites, whereas others (*An. quadriannulatus*, *An. bwambae*) are poor carriers, while still others (*An. merus* and *An. melas*) are intermediate. Thus knowledge of which of these morphologically identical species prevails in a location allows assessment of the threat they pose. It is not always clear if the relative importance of these sibling species is actually due to the relative physiological ability to support development of the parasite (competence) or other behavioral (e.g., host preference) or ecological factors (not living closely associated with human habitats). Regardless, the various subunits of *An. gambiae* s.l. pose very different public health threats.

On the intraspecific level, there is also evidence of considerable variation in ability to transmit pathogens among individual genotypes. *Aedes aegypti* is the best studied in this regard primarily because it is easy to rear in the laboratory and the primary vector of several viral diseases of contemporary concern. Table 1 shows results for three viruses. Different mosquito populations were studied in the same laboratory using identical methods, so pairs of populations in each study are comparable. Souza-Neto et al. [26] review many more such studies for several other strains of viruses and *Ae. aegypti* populations. In some cases, individual genetic factors have sufficiently strong effects on competence they have been identified and mapped [27,28,29]. Lambrechts et al. [30] demonstrated local adaptation between *Ae. aegypti* populations and dengue virus isolates from their localities.

**Figure 4:**
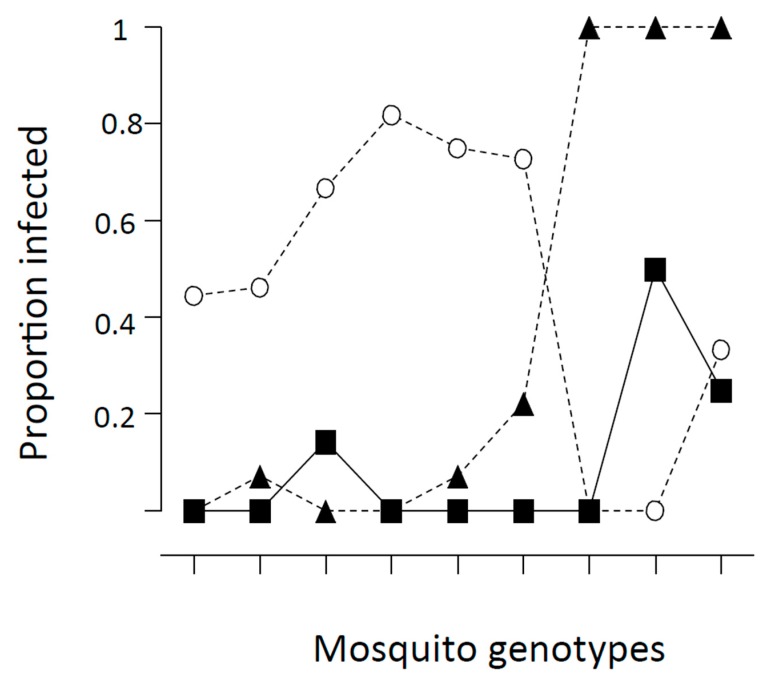
Proportion of *Anopheles gambiae* females infected after taking *Plasmodium falciparum*-infected blood meals. Three lines are for three different isolates of the parasite, nine ticks along the X-axis represents nine different families of the mosquito. From [30,34].

While not so easily studied in the laboratory, *An. gambiae* s.s. has been shown to have similar variation in ability to be infected with *Plasmodium falciparum*. Figure 4 shows the results of infecting nine different families of *An. gambiae* derived from the same population from Kenya for three different isolates of the malaria parasite (also from Kenya) [30]. Obviously, there is considerable variation even *within* a mosquito population for susceptibility to infection and different Plasmodium isolates also vary considerably, confirming that heterogeneity of the pathogen is important in this regard.

*GWAS*: A relatively new methodology in genetic analyses has arisen as the result of advances in acquiring genome-level data (reviewed in [35]). It is now possible to identify genes responsible for different phenotypes, i.e., bridge the genotype-phenotype chasm, even in non-model organisms that are not easily reared in the laboratory. This method is called GWAS genome wide association studies. If one has a dense enough set of polymorphic markers (usually single nucleotide polymorphisms, SNPs) distributed along a genome in samples that differ in a phenotype, it is possible to make statistical associations of the SNPs to the phenotypes. SNPs exhibiting such associations are unlikely to be directly causally related to the phenotype of interest, but rather they are non-randomly linked to the causative genetic factors. Thus the degree of non-random association between physically linked positions in a genome (called linkage disequilibrium, LD) is important in determining the success of this methodology. Too little LD means extremely high density of markers and very large sample sizes are needed to detect significant associations, too much LD means any association will mark such a large region of the genome as to be of little guidance as to number or identity of candidate causative genes.

**Figure 5:**
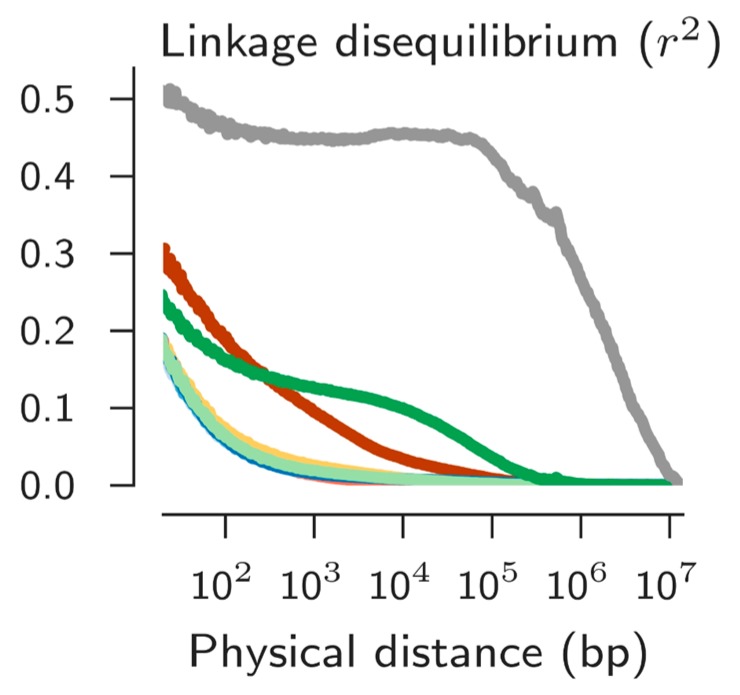
Linkage disequilibrium across populations of *An. coluzzii* and *An. gambiae* s.s. Four indistinguishable lines with lowest LD are from West Africa. Maroon line is *A. coluzzii* from Angola, dark green line is *An. gambiae* s.s. from Gabon, and the grey is *An. gambiae* s.s. from Kenya. From Reference [11].

Initial studies of *An. gambiae* and its sibling *An. arabiensis* reported very low LD in these species, generally not greater than extending over 200 bps [36,37]. Such observations would discourage trying to perform GWAS with these important vectors. However, the recent complete genome sequences of 765 individuals of these species across Africa revealed the existence of significant LD is some populations (Figure 5). Like so many other traits, LD is not a set parameter in a species. Importantly, this means GWAS may well be feasible if one chooses the right population to study. In fact GWAS has been successful in this group of mosquitoes for at least two traits, insecticide resistance [38] and desiccation resistance [39].

A similar situation occurs for *Ae. aegypti*. The level of LD found in populations is expected to be affected by the number of individuals that found a population (affecting effective population size) and the age of the population. Given the history of *Ae. aegypti* [17,40] it is expected that the ancestral populations that still exist in Africa should have less LD than populations outside Africa. Africa populations are relatively old and, at least historically, their habitat (tropical forest) was continuous implying large populations. Outside Africa, populations are likely to have been started with relatively few individuals, exist in discontinuous habitats, and are relatively young, not more than 500 years old, many much younger. One measure of LD is the distance at which the maximum observed correlation between loci drops to one-half. In a population from Mexico this occurs at 198 Kb while in an African population (Gabon) this occurs at 13 Kb [29].

A basic feature of genomes closely related to LD is recombination rate. When comparing two well-studied vectors, *An. coluzzii* and *Ae. aegypti*, we see a considerable difference in recombination rate per physical distance. Recombination in *An. coluzzii* is about 1.3 cM/Mb [41] while for *Ae. aegypti* it is 0.33 cM/Mb [29], a *four-fold difference*.

Thus, like so many other traits, there is no single level of LD for a species, it varies among populations dependent primarily on age and size of populations. At least between species, rates of recombination also varies considerably directly affecting levels of LD. 

*Origins of New Introductions*: Many vectors are invasive species [42], species that have ongoing shifts in their distributions. These are often species closely associated with human habitats which enhances their spread by human activities as well as increasing their medical importance by being in close proximity to humans. *Aedes aegypti* is a classical well-studied invasive vector. In the last ~600 years it has spread from its native Africa around the tropical and subtropical world and continues to expand its distribution [43]. Due to the population-specific genetic heterogeneity of this species (Figure 3), it is possible to trace the origin of new introductions or what are thought to be new introductions.

Table 2 summarizes the power of using microsatellites or a battery of ~16,000 SNPs from a SNP chip [44]. Three studies where these methods have been applied are for recent introductions into The Netherlands [45], Washington, DC [46], and Las Vegas [47].

**Figure 6:**
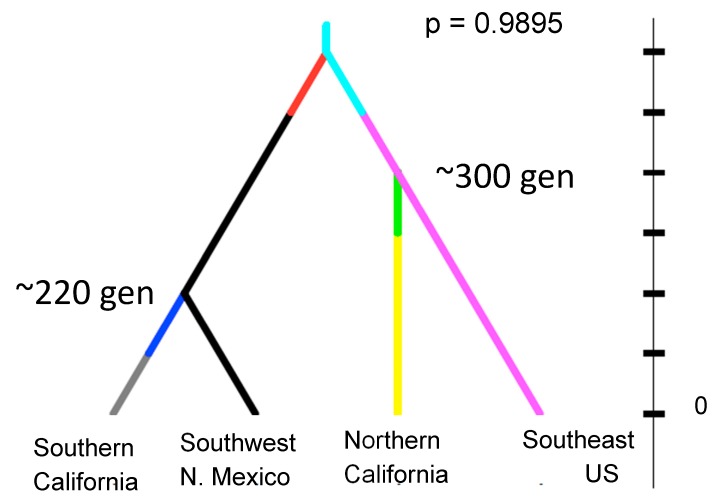
Estimates of the time of splits (in generations) of populations of *Aedes aegypti* in major regions of western North America based on microsatellites. The topology of the tree is supported over alternative topologies by the Bayesian posterior probability shown (methods in [51]). From [50].

Perhaps the most revealing and potentially disturbing example of genetic analyses of presumed new introductions is California. *Aedes aegypti* was first reported in California in 2013. Initial work on samples from northern California indicated a likely origin as the southeast of the US, possibly New Orleans [49]. Subsequent collecting in California revealed the species to be quite widespread including in southern California. It became clear that the northern populations closely related to the southeast US are genetically distinct from southern California populations that are closely related to southwest US populations (Arizona and New Mexico) and northern Mexico [50]. The disturbing result from these genetic analyses is that estimates of the age of the populations in California date to ~300 generations ago (Figure 6). If we assume about ten generations per year, possibly fewer given the relatively cool temperatures in northern California, this implies the populations have been there at least 30 years, much before their “discovery” in 2013. California has an extensive and active mosquito surveillance program, yet did not detect this species.

Similar to California where previously undetected populations are reported as new introductions is the case of the Black Sea. Beginning in the early 2000s, *Ae. aegypti* was reported as new introductions after an apparent absence of about 50 years (e.g., [52]). Genetic analyses of these “new” introductions showed them to be older than all other Asian populations dating to about 200 years [48].

*Inbreeding*: For many studies of genetics of vectors, e.g., genome sequencing and assembly, reducing genetic variation is desirable. The usual way this is done is to inbreed, produce lines from matings of close relatives. In many vectors this has proven problematic. 

One clear example is the easily-reared *Ae. aegypti* which is quite amenable to most genetic work. However, when lines with reduced genetic variation are attempted to be derived through inbreeding, they are only partially successful. One recent example is Powell and Evans [53]. Heterozygosity of the founding populations is expected to be reduced to (0.75)^n^ after n generations of full-sibling inbreeding, 13% after seven generations. The observed reduction after seven generations of full-sib matings was only to 72% of the starting heterozygosity. Similar results have been observed in other Aedes [54] and Anopheles [55,56].

This implies the genetic variation in natural populations of vectors imparts high fitness through such mechanisms as heterosis or overdominance; the expected frequencies of homozygotes are not obtained due to low fitness. This means the genetic variation so ubiquitous in vector populations is adaptive and almost certainly accounts for their ability to occupy diverse niches (e.g., reference [57]) and to respond quickly to evolutionary challenges (see next section). Reduction in fitness due to loss of genetic variation is almost inevitable with laboratory rearing and colonization of vectors. This can affect fitness traits in males with implications for genetic modification programs using releases of males [55].

## 4. Importance of Vector Variation in Control Programs

Controlling diseases through control of vector populations can be quantitative or qualitative. Quantitative control means reducing the number of vectors, qualitative control means genetically changing vectors to be less dangerous to humans. The standing genetic variation in vector populations plays a big role in determining the effectiveness of both approaches. 

*Quantitative control*: Quantitative control methods may reduce either the absolute numbers of vectors or frequency of vectors biting human hosts. Destruction of habitat, usually larval habitats, is well known and often effective. Screens and bed nets are effective especially for nighttime biters like many of the most important anopheline vectors of malaria. While genetic variation in vector populations may affect such control programs, for example, due to the propensity of different genotypes to take blood meals indoors or outdoors [58], it is much more obvious how genetic variation affects control programs based on insecticides and release of sterile vectors. 

Wherever insecticides have been used, resistance almost invariably arises. Resistance is most often due to genetic changes in populations by increasing *already present genetic variants* that allow individuals to survive the chemicals being applied. This is exactly the expectation of the populational view of diversity: So much variation exists in natural populations that almost any trait can be selected.

Less well appreciated is the role genetic variation in populations plays in sterile insect release (SIT) programs. This method has had limited application to vectors and more experience comes from agricultural applications. One issue directly related to heterogeneity of vectors is evolution of mating discrimination against the release strain. Because females that mate with released sterile males leave no offspring, there is strong selection favoring females that do not mate with the released males. Over time, the release strain males lose their effectiveness to suppress the population. This was documented in the Mediterranean fruitfly SIT control program [59]. While not documented in the longest such program, that for the screwworm, this may be due to the frequent changing of the release strain, some 17 times over the about 60 years [60].

**Figure 7:**
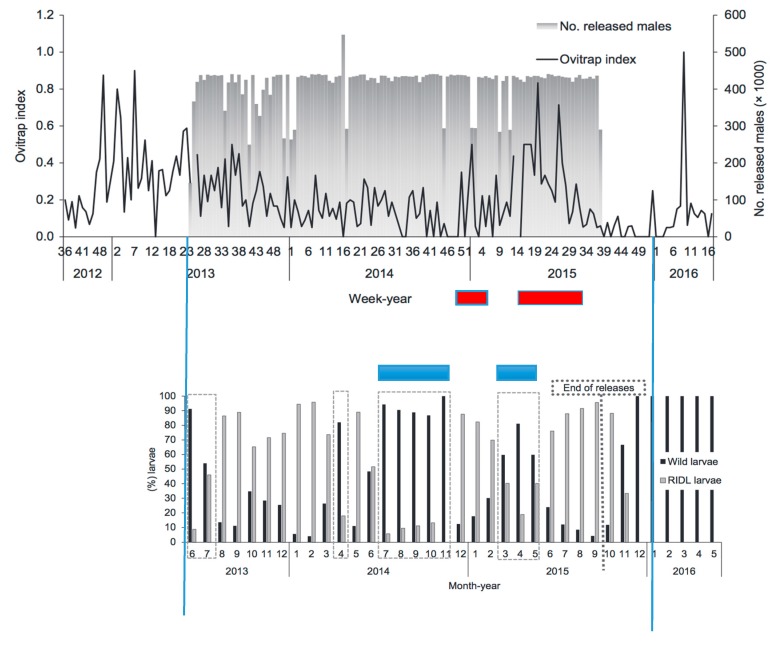
Results of releasing *Aedes aegypti* males carrying a dominant lethal gene in Jacobina, Bahia, Brazil. Blue lines were added to line up the same time in the two graphs taken from [63]. (**Upper graph**) shows the numbers released each week (grey bars, right scale). Dark line in upper graph is the ovitrap index as a measure of fertile female adults present. (**Lower graph**) shows the frequencies of wild type larvae reared from eggs in ovitraps (dark black lines) and F_1_ offspring from mating with the RIDL release strain (grey lines).

The most extensive SIT program for a vector of human diseases has been for *Aedes aegypti* employing a release strain with males carrying a dominant lethal gene, a method dubbed RIDL (Release of Insects with a Dominant Lethal) [61,62]. The most intensive releases have been done in Brazil, in particular in a city in Bahia, Jacobina. This release program began to lose its effectiveness after about 18 months of releases, Figure 7 [63]. Blue bars have been added in this figure indicating when released males had significantly reduced effectiveness in producing F_1_ eggs followed (lower graph), temporally, by the red bars when the adult female population significantly increased (upper graph). While not explicitly tested, this pattern is consistent with mating discrimination against the released males as a likely explanation for this breakdown of the effectiveness of control of numbers. 

*Qualitative control*: The idea of genetically altering vectors to render them less harmful to humans has a long history dating to at least 50 years. This requires two major steps. (a) Identifying a genetic variant (some DNA change) that results in a vector phenotype that is less capable of transmitting a pathogen. This may take the form of causing a physiological change that reduces or eliminates the growth of the pathogen in the vector, or other trait such as reducing or eliminating a preference for taking blood meals from humans. (b) The second step is to somehow replace the target vector population with the variant. Many studies have identified genetic changes that satisfy the first step, the major stumbling block has been the second step. To date no such program has been successful.

Despite this lack of progress, new optimism and hope arose with the discovery of the CRISPR/Cas9 system that seemed to hold great promise (reviewed in Reference [64]). In this system, DNA regions heterozygous for the CRISPR/Cas9 endonuclease convert the alternative allele into itself. This produces a distinct advantage to the spread of the gene through a population. Linking this system to a desirable allele (e.g., one conferring resistance to transmitting a pathogen) to the endonuclease would “drive” the resistant gene construct through a population. In theory, this should work very efficiently [65,66].

Unfortunately, there is increasing evidence that the genetic variation inherent in natural populations of vectors is likely to frustrate any such CRISPR/Cas9 type replacement programs. Both theory [67] and experimental data in laboratory populations of insects, Drosophila [68] and Tribolium beetles [69], indicate that resistance to the drive mechanism is very likely to arise quickly in any such program. This is due to a combination of naturally existing variation in the target site for the endonuclease as well as the fact that the endonuclease itself is mutation-inducing with error prone repair where it cuts, leading to destruction of the target site.

While not a modification of a vector’s genome, there is a successful modification of a vector’s microbiome that renders the carriers less efficient vectors of human diseases. This is based on the endosymbiotic bacteria, *Wolbachia*. This bacterium is ubiquitous in insects [70] but does not exist naturally in *Ae. aegypti* [71]. However, when artificially infected with *Wolbachia* in the laboratory, *Ae. aegypti* become less efficient in transmitting dengue virus and other pathogens [72]. Release of *Ae. aegypti* with Wolbachia has led to establishment in the field [73]. While the infection remains stable where locally established, so far it has only spread at a very slow rate [74], much less than in other insects like Drosophila [75]. In another locality (Rio de Janeiro, Brazil) release of the same strain derived from Australian mosquitoes did not establish, possibly due to it being particularly susceptible to the insecticide being used locally. Outcrossing to native Rio *Ae. aegypti* produced a strain that could establish in that city (L. Moreira, personal communication). This is strong evidence that any attempts to genetically modify populations should be based on the genotypes prevalent in the target population.

Finally, there is one proposed method of genetic control of vectors that takes advantage of the genetic diversity inherent in populations. This is to select from the natural population to be controlled for a strain or strains of mosquitoes refractory to transmission of a pathogen [76]. By releasing such strains repeatedly, the target population could be reduced significantly in competence. Because no new genetic material is introduced, just increasing frequencies of already existing refractory alleles, this method has been called “genetic shifting”. Recent modeling has indicated that size and number of releases needed to significantly lower the ability of the target population to transmit are realistically achievable [77]. Because the release strain is identical to genotypes already present, mating discrimination is less likely to evolve and if it does, new strains for release could be re-selected from the target population. The replacement of the release strain is much less laborious than reconstructing transgenic modified strains.

## 5. Concluding Remarks

The forgoing is necessarily biased by the author’s familiarity with the field and has been confined to mosquito examples for the most part. Nevertheless, the major point should be clear: It is safe to conclude that arthropod vectors in general harbor large amounts of genetic variation that affect both how research is carried out and what control programs are most likely to be effective. While it may seem that I have set up a “straw man” and that the points explicated here are obvious, much vector biology research still proceeds with typological concepts at the forefront. I have purposely not cited such work to avoid irritating colleagues. Suffice it to end with one anecdote. Mario Coluzzi spent his life promoting populational thinking in vector biology. Near the end of his life (in 2012), in referring to a very prominent vector biologist, he asked me “Do you think he finally gets it?”.

## Figures and Tables

**Table 1:** Examples of vector competence studies on *Aedes aegypti* for three of the major viruses this species transmits, Zika, dengue, and yellow fever. The geographic origin of mosquitoes tested is in first column with the virus in the second. Infection rate (third column) is the percent of females that blood fed on infective blood that became infected.

Geographic Origin Mosquito	Virus	Infection Rate	Reference
Salvador, Brazil	Zika DAK AR	100%	[31]
Rio Grande, Texas	“	40%	
Singapore	Dengue Guinea C	90%	[32]
Bangkok	“	10%	
Guatemala	Yellow Fever Asibi	2%	[33]
Kwa Dzivo Kenya	“	57%	

Note that in this table are presented studies using the same strain of virus and assayed in.

**Table 2:** Probabilities of correctly assigning an individual *Aedes aegypti* to its population of origin using either microsatellite variation or ~16,000 SNPs from a chip. From [48].

Genetic Data Type	Probability of Assignment to Correct Continent	Probability of Assignment to Correct Population
Microsatellites	99.9%	80.9%
SNPs from chip	100%	99.7% *

* An exception is Florida and Costa Rica which are not distinguishable.
